# Optimization and Simulation on Gas Flow and Temperature Fields on the Homoepitaxial Growth of N-Doped 4H-SiC Wafers

**DOI:** 10.3390/mi17030305

**Published:** 2026-02-28

**Authors:** Guoliang Zhang, Tiantian Li, Yingbin Liu, Jinfeng Sun, Shaofei Zhang

**Affiliations:** 1 Hebei Key Laboratory of Flexible Functional Materials, School of Materials Science and Engineering, Hebei University of Science and Technology, Shijiazhuang 050018, China; zhangguoliang@poshing.cn (G.Z.); sjf301@126.com (J.S.); zhangshaofei988403@163.com (S.Z.); 2 Hebei Poshing Electronics Technology Co., Ltd., Shijiazhuang 050200, China

**Keywords:** N doping concentration, uniformity, CVD parameters, mechanism of site competition epitaxy, simulation

## Abstract

The uniformity of nitrogen (N) doping concentration in 4H-SiC epitaxial wafers is a critical determinant of electrical consistency and device reliability. In this study, key chemical vapor deposition (CVD) growth parameters, including the C/Si ratio, H_2_ carrier gas flow rate, flow split ratio, and growth temperature, were systematically adjusted to investigate their effects on the N doping concentration and uniformity of 6-inch 4H-SiC homoepitaxial layers. The relationships between these parameters and characteristic phenomena such as site-competition epitaxy, along-track depletion of carbon source, and the distinct “W-shaped” doping profile were comprehensively analyzed. Furthermore, simulations of the flow and temperature fields within the reaction chamber and across the SiC epitaxial wafer revealed that under optimized conditions a stable parallel flow field forms above the wafer, accompanied by a uniform temperature distribution, thereby creating an ideal environment for homogeneous N doping. This work provides both theoretical insight and practical guidance for enhancing doping uniformity in large-size SiC epitaxial wafers.

## 1. Introduction

Silicon carbide (SiC), owing to its superior physical properties including high breakdown electric field, high thermal conductivity, and high electron saturation drift velocity, has become an ideal substrate material for high-voltage and high-power electronic devices [1,2,3]. Among various polytypes of SiC, 4H-SiC exhibits a hexagonal crystal structure with ABAC stacking sequence, offering both high carrier mobility and excellent crystal stability, which makes it the preferred choice in industrial applications [4,5,6]. Nevertheless, the performance of devices based on 4H-SiC fundamentally depends on the quality of the epitaxial layers, among which the doping concentration and its uniformity are critical parameters. For n-type doping, nitrogen (N) is the most commonly used dopant, which provides conduction electrons by substituting carbon sites in the lattice. Previous study has shown that controlling the uniformity of N doping within 4% is essential for producing high-performance devices, including avoiding localized electric field crowding, facilitating uniform carrier transport in the drift region and JFET region, promoting homogeneous distribution of carrier mobility in the channel region, and so on [7]. Feng et al. [8] demonstrated that appropriately increasing the doping concentration while maintaining the breakdown voltage can significantly reduce the specific on-resistance and effectively suppress capacitive feedback, thereby improving the high-frequency figure of merit (HF-FOM) and overall robustness of SiC MOSFETs. A recent study has also revealed that a uniform doping concentration is beneficial for enhancing the interfacial and electrical characteristics of 4H-SiC MOS capacitors [9]. However, achieving uniform nitrogen doping remains a significant challenge, largely owing to the complexity of the chemical vapor deposition (CVD) epitaxial growth process. This process involves coupled multi-physics and multiscale dynamics, encompassing gas-phase transport, surface reactions, and heat transfer.

From the perspective of gas-phase transport, the flow distribution of precursors (e.g., silicon and carbon sources) and the N_2_ within the reaction chamber determines the flux uniformity delivered to different regions of the substrate surface [10,11,12]. A non-uniform flow field can lead to varying supply rates of reactants along the wafer radial direction, resulting in spatial inhomogeneity of N distribution [13,14]. Moreover, CVD reaction kinetics are strongly influenced by growth parameters. Larkin’s “site-competition epitaxy” theory indicates a competitive relationship between N and lattice carbon (C) or silicon (Si) atoms during epitaxy, suggesting that any factors altering the C/Si ratio, such as carrier gas flow rate and growth temperature, affect the uniformity of N doping concentration and uniformity [15,16]. Significant progress has been achieved by optimizing individual parameters. For instance, Inoue et al. [17] investigated the dependence of N incorporation on step-flow velocity by controlling nitrogen doping concentrations (from ~10^17^ cm^−3^ to 10^19^ cm^−3^), and their analysis using a two-site exchange model revealed improved doping uniformity with increasing nitrogen concentration. Notably, few studies have systematically examined the underlying influence mechanisms of flow field and temperature field distributions on the uniformity of N doping in SiC epitaxial wafers.

In light of this, this study proposes a synergistic optimization strategy combining process control with simulation to achieve theoretical insights and practical improvement in N doping uniformity. Through systematic adjustment of key CVD epitaxial growth parameters, including the C/Si ratio, carrier gas flow, and growth temperature, the optimal parameter for enhancing N doping uniformity was obtained. Subsequently, two-dimensional simulations were performed to establish the relationships between process parameters and the resulting flow and temperature fields inside the chamber. The experimental results indicate that a C/Si ratio of 0.95, a proper H_2_ gas flow rate of 100 slm, and a growth temperature at 1610 °C yielded an N doping uniformity of 3.35%. Simulations under these conditions revealed a stable, parallel gas flow above the wafer surface, forming a uniform and well-defined concentration boundary layer. Moreover, the temperature distribution across the SiC surface showed a small variation, providing a favorable environment for uniform N doping. This study offers theoretical guidance for improving doping uniformity in large-size SiC epitaxial wafers and contributes to enhancing wafer yield and reliability.

## 2. Materials and Methods

### 2.1. Materials

High-purity ethylene (C_2_H_4_, 99.999%), nitrogen (N_2_, 99.999%), and trichlorosilane (SiHCl_3_, 99.999%) were purchased from NEWRADAR. High-purity hydrogen (H_2_, 99.999%) was prepared by first generating H_2_ through water splitting, followed by drying and purification. The 6-inch 4H-SiC substrate used in the experiments was purchased from Hebei Synlight Semiconductor Co., Ltd.

### 2.2. Synthesis of N-Doped SiC

**Figure 1:**
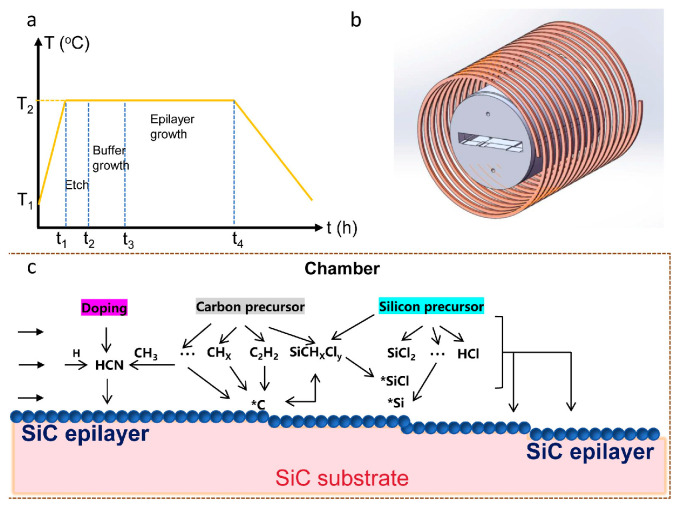
(**a**) CVD growth process along with time variation, including etching, buffer layer growth, and epitaxial wafer growth. (**b**) The geometric configuration of the growth zone in the CVD furnace. (**c**) The homogeneous epitaxial chemical reaction with N doping on SiC substrate.

A PE106-type horizontal hot-wall reactor (Beijing NAURA Technology Group Co., Ltd.) was used for growing the SiC epitaxial wafer. The 6-inch 4H-SiC substrate was placed on a graphite heating susceptor for homoepitaxial growth. H_2_ served as the carrier gas (varied from 90 to 110 slm) and C_2_H_4_ and SiHCl_3_ acted as the C and Si sources, respectively. N_2_ was used as the dopant and the flow rate fixed at 200 slm. The SiC epitaxial wafer was grown via CVD by adjusting growth parameters, with a chamber pressure of 10,000 Pa. The C/Si ratio was controlled by adjusting the flow rates of C_2_H_4_ and SiHCl_3_, while the flow rate of the C_2_H_4_ gas was fixed at 200 slm. The temporal evolution of the CVD growth process, spanning a total duration of approximately 90 min, is depicted in Figure 1a. Prior to introducing the C and Si precursors, in situ HCl etching was performed at the growth temperature (T_2_) to remove surface-damaged regions. Subsequently, a buffer layer was grown for 10 min to further suppress the propagation of substrate defects and improve the epitaxial layer quality. Finally, the epitaxial layer was grown for about 10 min at a growth rate of 60 μm·h^−1^, yielding a controlled thickness of 10 μm for the SiC epitaxial wafer in this study.

### 2.3. Calculation of N Dopant Concentration and Uniformity

Capacitance-voltage (C-V) profiling with Hg Schottky contacts was used to detect the N doping concentration distribution of 6-inch 4H-SiC epitaxial wafers. For each SiC epitaxial wafer, thirteen-point concentration measurements were performed. The variance (σ), average value (*mean*, cm^−3^), and uniformity (δ) of the *N* doping concentration were then calculated using the equations below [18,19,20]:

(1) $$\sigma =\sqrt{\left(\sum_{i=1}^{n}\left(x_{i}-m\right)/(N-1)\right)}$$

(2) $$mean=\sum_{i=1}^{n}x_{i}/N$$

(3) $$\delta =\frac{\sigma }{mean}\cdot 100\%.$$

where *x_i_* is the *N* doping concentration, cm^−3^; *N* is the number of measurement points.

### 2.4. Simulation

First, a multi-physics coupled geometric model of the reaction chamber was established based on the structure of reactor and operating conditions, as shown in Figure 1b. According to the actual chamber design, the SiC wafer was positioned in the center of the chamber during the simulation. This region provides a growth environment with uniform temperature and stable gas flow. Simultaneously, the SiC wafer rotates continuously at a low speed driven by an air-floating graphite holder to achieve more uniform growth conditions.

Given the thin thickness of the SiC epitaxial wafer, the influence of gas flow variations perpendicular to the epitaxial wafer on the concentration uniformity can be neglected [21]. Therefore, a two-dimensional field model was adopted, and COMSOL software was used to simulate the gas flow and temperature field distribution during the SiC epitaxial growth process [22]. The gas in the reactor was treated as an ideal gas, and the gas flow state was laminar. Since the SiC epitaxial growth involves multiple physical fields, including electromagnetic, flow, and temperature fields, the governing equations in the COMSOL laminar flow module were Maxwell Equations (4)–(7) [23], the continuity Equation (8) [24], the mass conservation Equation (9) [25], and the momentum conservation Equation (10) [26].

(4) $$\nabla \cdot E=\frac{\rho_{q}}{\varepsilon_{0}}$$

(5) ∇·B=0

(6) $$\nabla \times E=-\frac{\partial B}{\partial t_{0}}$$

(7) $$\nabla \times B=\mu_{0}J+\mu_{0}\varepsilon_{0}\frac{\partial E}{\partial t}$$

(8) $$\frac{\partial \rho }{\partial t}+\nabla (\rho \cdot \mu )=0$$

(9) ρ∇·u=0

(10) ρ(u·∇)u=∇·[−pl+K]+F

where ρ is the mass density of the fluid, g L^−1^ or slm; u is the fluid velocity, m s^−1^; p is the static pressure of the fluid, Pa; l is the second-order unit tensor; K is the deviatoric stress tensor; and F is the volumetric force density, N m^−3^.

## 3. Results

### 3.1. N Doping Mechanism During CVD Growth 4H-SiC Epitaxial Wafer

The mechanism for the CVD growth of N-doped 4H-SiC wafers in a H_2_ carrier gas environment is illustrated in Figure 1c. At a high temperature of SiC epilayer growth, C_2_H_4_ decomposes into C_2_H_2_ to provide active C atoms (*C), while SiHCl_3_ undergoes pyrolysis to generate SiCl_2_, supplying active Si atoms (*Si). These species undergo complex chemical processes on the substrate surface via a step-flow growth mode, including adsorption, deposition, and desorption. Simultaneously, the introduced N_2_ is converted into HCN during CVD, which serves as the primary source for N incorporation, thereby completing doping in SiC epitaxial wafers. The incorporation of N dopants is explained by the site-competition epitaxy mechanism, whereby N atoms compete with carbon sources for lattice sites during the growth of SiC [27]. This process is governed jointly by the temperature and flow fields, both of which are adjustable through growth parameters.

### 3.2. Effect of C/Si Ratio

**Figure 2:**
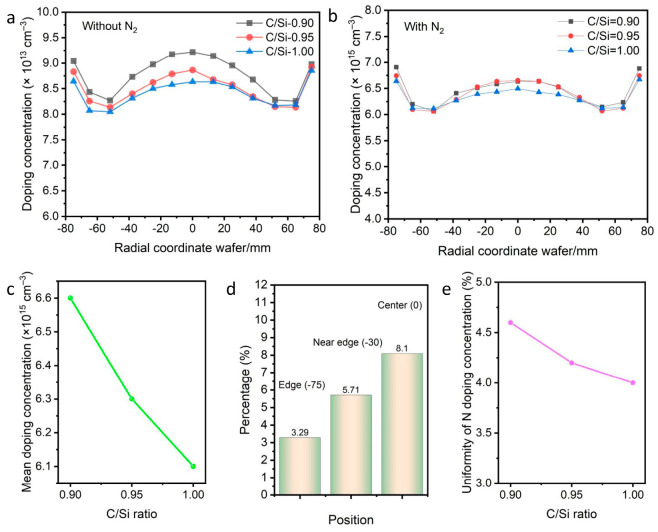
Doping concentration versus C/Si ratio in SiC epitaxial wafers without (**a**) and with N_2_, (**b**) flow, (**c**) Mean doping concentration versus C/Si ratio in SiC epitaxial wafers with N2 flow, (**d**) Radial reduction in concentration at specific positions, (**e**) Uniformity of N doping concentration.

The background N dopant concentration was first examined at different C/Si ratios without introducing N_2_ flow under a growth temperature of 1620 °C. As shown in Figure 2a, a measurable level of N incorporation was still observed in the SiC epitaxial wafer even in the absence of intentional N_2_ supply into the reaction chamber. The N concentration varied with the C/Si ratio, reaching a maximum of 9.21 × 10^13^ cm^−3^ at a C/Si ratio of 0.9. The occurring N mainly originate from residual N sources inherent in the reaction chamber or the graphite susceptor inevitably, which become activated under high temperature conditions and subsequently incorporate into the SiC epitaxial wafer as dopants. To improve the N doping concentration, introducing N_2_ gas flow during CVD growth can significantly increase the doping level, and the uniformity can be optimized by controlling the growth parameters. Given the competition between N atoms and C sources for lattice sites, modifying the C/Si ratio is crucial. Figure 2b shows the variation of N doping concentration as the C/Si ratio increases from 0.90 to 1.00 under fixed growth conditions of 1620 °C with adding N_2_. A “W-shaped” profile was clearly observed. By measuring, the mean doping concentrations at C/Si ratios of 0.90, 0.95, and 1.00 reach 6.6 × 10^15^ cm^−3^, 6.3 × 10^15^ cm^−3^, and 6.1 × 10^15^ cm^−3^, respectively (Figure 2c). It is evident that under a constant N_2_ flow, the N doping concentration declines along with increasing the C/Si ratio. This is primarily attributed to a higher C/Si ratio favoring the competition of N atoms against C sources for lattice sites, demonstrating a distinct “site-competition epitaxy” mechanism [27]. Furthermore, at the C/Si ratio of 0.95, the mean doping concentrations at the wafer edge, near edge, and center decreased by approximately 3.29%, 5.71%, and 8.10%, respectively (Figure 2d). Figure 2e displays the measured doping uniformity. Clearly, the doping uniformity value reaches 4.1% at a C/Si ratio of 0.95, much lower than the wafer at C/Si ratio of 0.90 (4.6%), but a little higher than the wafer at C/Si ratio of 1.00 (4.0%). This phenomenon confirms that the C source exhibits a higher “along-track depletion” rate compared to the Si source. Therefore, increasing the C/Si ratio can compensate for the higher depletion of C sources toward the wafer center, which helps improve doping uniformity. However, an excessively high C/Si ratio may lead to increased step bunching and the formation of triangular defects, adversely affecting epilayer quality [28].

**Figure 3:**
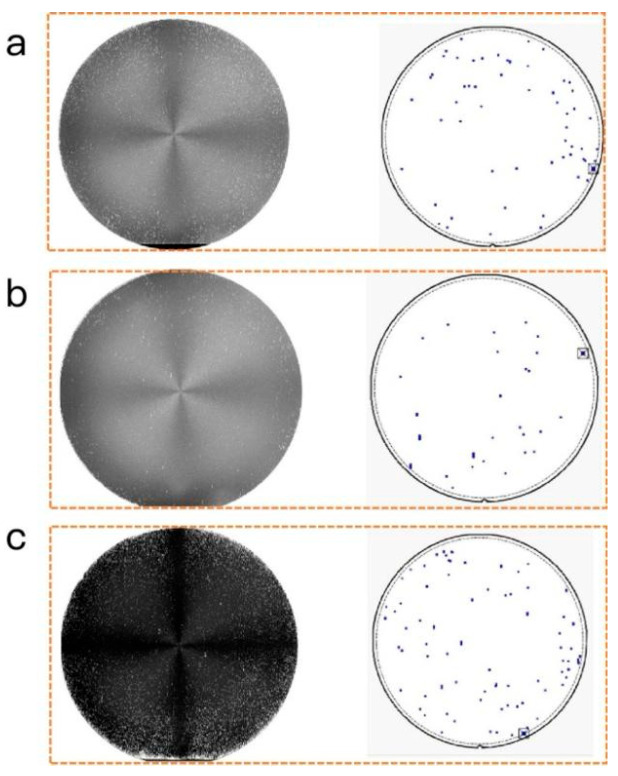
Defect characterization for C/Si ratios of 0.9 (**a**), 0.95 (**b**), and 1.00 (**c**).

The distribution of various defects is summarized in Figure 3 and Table 1. At C/Si ratios of 0.90, 0.95, and 1.00, the triangular defect counts were 25, 8, and 38, respectively. In comparison, step defects numbered 12, 14, and 35, whereas the counts for basal plane dislocations (BPDs) were 21, 6, and 4, respectively. By comparison, a C/Si ratio of 0.95 resulted in the minimum total defect count of 28. The increase in defect count with C/Si ratio is primarily due to non-stoichiometric conditions arising from ratios that are either too low or too high, which lead to deposition-related defects. Therefore, based on a comprehensive evaluation of doping uniformity and material quality, a C/Si ratio of 0.95 is determined to be optimal for the SiC epitaxial wafer growth.

### 3.3. Influence of Carrier Gas Flow

**Figure 4:**
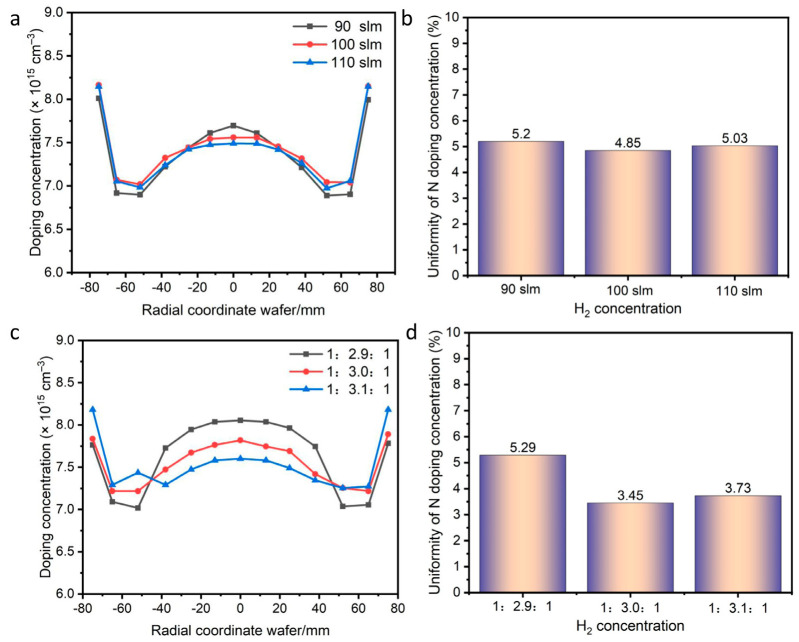
Doping concentration and uniformity versus key process parameters. (**a**,**b**) H_2_ flow rate; (**c**,**d**) flow split ratio.

With a fixed C/Si ratio of 0.95, the effect of H_2_ carrier gas flow rate on doping concentration and uniformity was investigated. As shown in Figure 4a, the doping concentration profile across the SiC epilayer exhibits a “W” shape at H_2_ flow rates of 90, 100, and 110 slm. Moreover, the average N doping concentration varies significantly with changes in the H_2_ gas flow. The reason is that, under constant process pressure, an increase in H_2_ gas flow may lead to a decrease in the reactor temperature and an increase in the gaseous H_2_ concentration. This slows down the depletion of source gases, particularly the C source, along the gas stream. Consequently, the optimal doping uniformity of 4.85% was achieved at an H2 flow rate of 100 slm, compared to 5.20% and 5.03% at 90 and 110 slm, respectively (Figure 4b). This suggests that a proper H_2_ carrier gas flow rate may enhance convection and heat transfer, while the adsorption/reaction of Si and C likely approaches saturation at a specific flow rate, yielding optimal N doping uniformity.

To investigate the relationship between doping uniformity and the gas flow field, the flow split ratio was varied as 1:2.9:1, 1:3.0:1, and 1:3.1:1 while keeping the total flow and C/Si ratio constant. Figure 4c shows the doping concentration profiles under different split ratios. As the central inlet flow increases, the doping concentration at the wafer center decreases while that at the edge increases. The smallest deviation between edge and center concentrations, along with the best overall uniformity of 3.45%, was observed at a split ratio of 1:3.0:1. At ratios of 1:2.9:1 and 1:3.1:1, the uniformity was 5.29% and 3.73%, respectively (Figure 4d). This confirms the significant impact of the flow split ratio on doping uniformity. A higher flow velocity results in a thinner boundary layer; regions with thinner boundary layers experience higher growth rates and consequently lower doping concentrations [29].

### 3.4. Influence of Temperature

**Figure 5:**
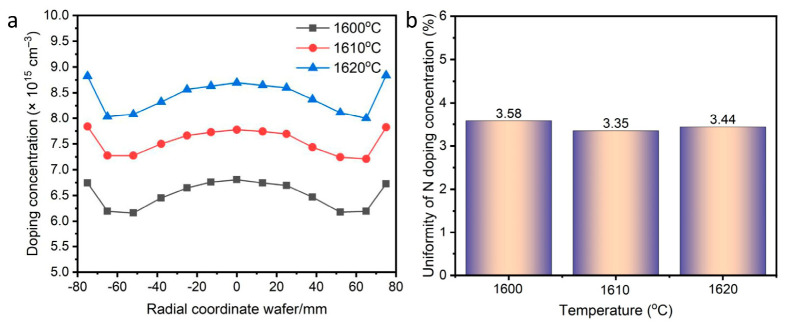
Doping concentration (**a**) and uniformity (**b**) at different CVD process temperatures.

Growth temperature is a fundamental parameter in SiC epitaxy. Selecting an appropriate temperature is crucial for achieving high N doping uniformity. In this work, the CVD growth temperature at 1600, 1610, and 1620 °C was conducted under specific C/Si and H_2_ flow conditions. Figure 5a presents the doping concentration profiles at different growth temperatures. The profiles exhibit a “W” trend across the wafer. The average doping uniformity was 3.35% at 1610 °C, superior to 3.58% at 1600 °C and 3.44% at 1620 °C (Figure 5b), indicating that 1610 °C is more conducive to improving uniformity. This is primarily because the adsorption, migration, and incorporation of N atoms into the SiC lattice require overcoming an energy barrier. At lower temperatures, the mobility of dopant atoms is poor, leading to their non-uniform aggregation at specific sites and causing micro- and macro-scale concentration fluctuations. Conversely, excessively high temperatures may induce unstable natural convection, disrupting the ideal laminar flow and leading to in-plane doping non-uniformity.

### 3.5. Simulation of Gas Flow and Temperature Fields

**Figure 6:**
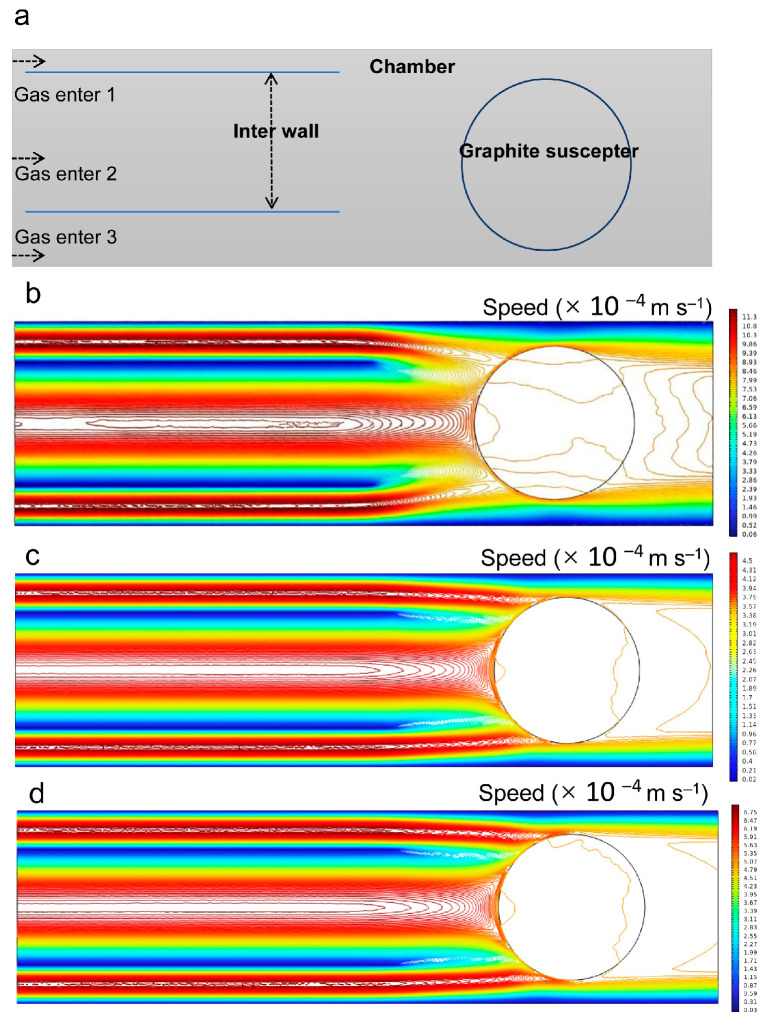
(**a**) Geometry of the CVD reactor with gas flow direction. Distribution of gas flow field in reaction chamber with different H_2_ gas flow rates: (**b**) 90 slm, (**c**) 100 slm, (**d**) 110 slm.

The “W”-shaped doping concentration profile observed in SiC epitaxial wafers is primarily attributed to the combined effects of gas flow dynamics, temperature field distribution, and reactant transport during epitaxial growth. To gain deeper insight into the correlation between doping uniformity and the gas flow field, simulations of the flow field during SiC epitaxial growth were performed. Figure 6a illustrates the geometry of the CVD reactor with gas flow direction. The H_2_ carrier gas flow was first simulated in the CVD reactor, including the graphite susceptor. At a low H_2_ flow rate of 90 slm (Figure 6b), the velocity contours over the wafer exhibit a complex pattern with pronounced gradients, indicating a flow regime dominated by viscous effects and non-uniform gas distribution, which leads to a higher N doping concentration at the edge. As the flow rate increases to 100 slm (Figure 6c), the contours become uniform and parallel, and the velocity difference between the edge and center regions is reduced. This optimum condition suggests the establishment of a stable, well-developed flow that enhances lateral uniformity, likely due to a thinned boundary layer and improved momentum transfer. However, a further increase to 110 slm (Figure 6d) disrupts this uniformity, resulting in a non-uniform contour pattern over the wafer. This degradation may be attributed to excessive flow momentum, which can induce flow instability, turbulence onset, or altered reactor temperature profiles, thereby compromising the ideal stagnation flow geometry achieved at 100 slm. Although the gas flow field was optimized by adjusting the H_2_ flow rate, the velocity near the edge regions remained relatively high. This locally enhanced flow increases the atomic step density at step edges and prolongs the residence time of surface-adsorbed species, ultimately resulting in a higher N doping concentration in those areas.

**Figure 7:**
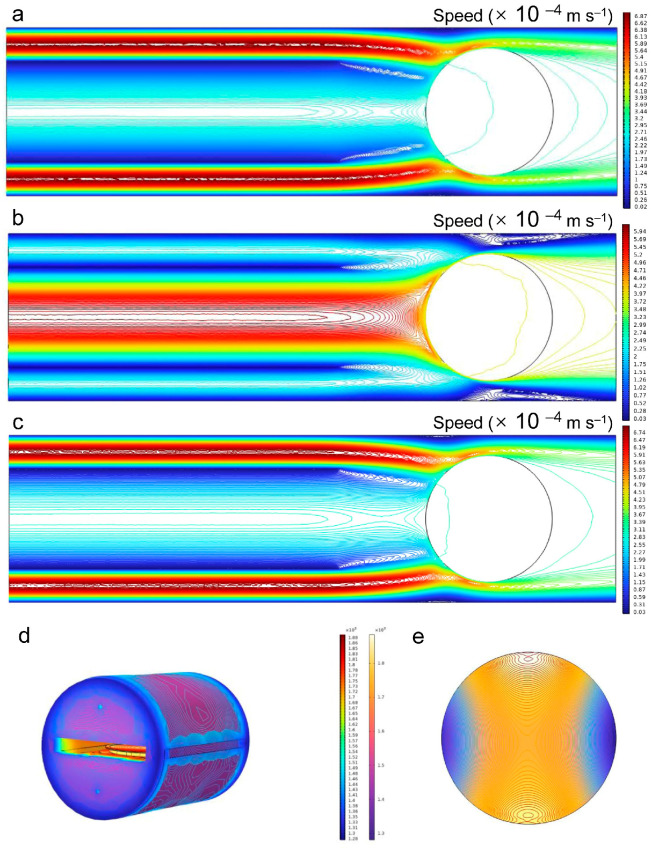
Distribution of gas flow field in reaction chamber with different H_2_ gas flow rates: (**a**) 30:40:30, (**b**) 20:60:20, (**c**) 10:80:10. Distribution of temperature field in reaction chamber (**d**) and on the SiC epitaxial wafer (**e**) at 1610 °C.

The effect of the flow split ratio on the velocity field was also examined via the three gas inlets of the CVD reactor geometry. Figure 7 presents the simulated velocity field distributions at a fixed total flow of 100 slm with different split ratios. With a flow split ratio of 30:40:30 across the gas inlets (Figure 7a), the resulting velocity contours over the wafer area exhibit significant non-uniformity. This uneven flow distribution is attributed to an imbalance in momentum injection from the inlets, which disrupts the formation of a symmetric stagnation flow above the wafer, leading to localized regions of higher and lower velocity, which may lead to a “W-shape” distribution of N doping concentration. The most uniform and widely distributed velocity contours are achieved with a 20:60:20 flow split (Figure 7b). In this regime, the flow field effectively promotes consistent reactant transport and temperature distribution across the wafer. In contrast, a 10:80:10 split (Figure 7c) reintroduces significant contour unevenness, degrading uniformity. This demonstrates that the uniformity of gas flow fields is highly sensitive to the flow split balance, with the 20:60:20 ratio representing an optimal point between the extremes of excessive side flow (as in 30:40:30) and an overly dominant center flow.

Given the aforementioned advantage of 1610 °C for uniformity, the temperature field within the chamber at 1610 °C was simulated, including temperature contours and surface temperature plots (Figure 7d). The contours are essentially symmetric about the geometric center. A temperature gradient exists between the interior and the edge of the graphite growth cavity, primarily dictated by the geometry. The outer region of the insulation layer remains cooler, while the growth zone reaches the target temperature. This thermal difference may contribute to a higher concentration of N dopants in the central region. However, with the rotation provided by the gas-foil susceptor, the temperature gradient across the SiC wafer surface is significantly reduced. The temperature field across the SiC epilayer is displayed in Figure 7e. It indicates a temperature difference of 19.3 °C between the internal and edge regions within the 6-inch SiC epitaxial wafer. The higher temperature in the central region accelerates step migration, increasing the flux of N atoms passing through step edges per unit time and thus enhancing the doping concentration. Consequently, the simulated distributions of temperature and gas flow affect the atomic step density at step edges and the residence time of surface-adsorbed species, making the N doping concentration as a “W-shape” across the wafer. On the one hand, the higher temperature in the central region is beneficial for improving the N doping concentration at this region. On the other hand, the fast gas velocity near edge of wafer leads to local fluctuations in the C/Si ratio, thus making N dopant enrichment. According to the “site-competition epitaxy” theory, the above temperature and gas flow fields result in elevated doping concentrations in both the central and edge regions.

## 4. Conclusions

In summary, we systematically optimized the N doping uniformity in 6-inch 4H-SiC homoepitaxial layers through the combined CVD approach and multi-physics simulation. The experimental investigation of key growth parameters revealed a clear interdependence between process conditions and the resulting N doping uniformity. The observed “W-shaped” doping distribution was elucidated through the synergistic effect of site-competition epitaxy and along-track depletion of carbon. Consequently, the optimal parameter yielded a superior N doping uniformity of 3.35%, accompanied by a significant reduction in epilayer defects. Simulations of the flow and temperature fields provided physical insight into the experimental trends. Under the optimized conditions, a stable laminar flow and a uniform temperature distribution were achieved, creating a favorable environment for consistent dopant incorporation. This work not only identifies a practical CVD growth for high uniformity N doping in 4H-SiC epitaxial wafers but also establishes a coherent link between CVD parameters and N doping mechanisms with gas and temperature field distributions.

## Figures and Tables

**Table 1:** Statistical analysis of defect density as a function of C/Si ratio.

	Defect Classification	Triangular Defects	Step Defects	BPDs	Total
C/Si Ratio					
0.9		25	12	21	58
0.95		8	14	6	28
1.0		38	35	4	77

## Data Availability

All the material conducted in the study is mentioned in article.

## References

[1] Yu J., Yu Y., Bai Z., Peng Y., Tang X., Hu X., Xie X., Xu X., Chen X. (2022). Morphological and Microstructural Analysis of Triangular Defects in 4H-SiC Homoepitaxial Layers. Cryst. Eng. Comm..

[2] Liu W., Tong Z., Zhang C., Han X., Wang R., Yang D., Pi X. (2025). Doping and Minority Carrier Lifetime Uniformity of 4H-SiC Homoepitaxial Layers: Role of C/Si Ratio Distribution. Sci. China Inf. Sci..

[3] Yang Z., Zuo Y., Wang X., Zhou H., Tang H., Zheng C., Zhang R., Tang Z., Dai K., Fan X. (2025). Nondestructive Analysis of Interface Damage and Stress in Al-Ion Implanted 4H-SiC Homoepitaxial Wafers via Micro-Raman and Multiscale Simulation. Appl. Surf. Sci..

[4] Tian J., Tang Z., Tang H., Fan J. (2025). Multi-Objective Optimization of 4H-SiC Homoepitaxy Chemical Vapor Deposition Process Fusing Support Vector Machine with Multiphysics Simulations. Mater. Today Commun..

[5] Zhao L.X., Yang L., Wu H.W. (2020). High Quality 4H-SiC Homo-Epitaxial Wafer Using the Optimal C/Si Ratio. J. Cryst. Growth.

[6] Zhao L. (2020). Surface Defects in 4H-SiC Homoepitaxial Layers. Nanotechnol. Precis. Eng..

[7] Chen Q., Yao Y., Zhang J., Li B., Che L., Zhang X., Fan H., Tian J., Peng Y., Xie X. (2025). Effect of Nitrogen Doping Concentration on 4H-SiC Laser Slicing. J. Am. Ceram. Soc..

[8] Feng J., Zheng L., Cheng X., Shen L., Zhou X., Lu W., Zeng J. (2025). Comprehensive Trade-Off Strategy for SiC MOSFETs Using Buried-MOS Configuration. Chip.

[9] Wang Q., Cheng X., Zheng L., Ye P., Li M., Shen L., Li J., Zhang D., Gu Z., Yu Y. (2017). Enhanced Interfacial and Electrical Characteristics of 4H-SiC MOS Capacitor with Lanthanum Silicate Passivation Interlayer. Appl. Surf. Sci..

[10] Ota T., Asano S., Inoue Y., Ohtani N. (2023). Experimental and Simulation Studies of Surface Segregation-Limited Nitrogen Incorporation at the Growth Front of Physical Vapor Transport-Grown 4H-SiC Crystals. J. Appl. Phys..

[11] Tsuchida H., Kanda T. (2024). Advances in Fast 4H–SiC Crystal Growth and Defect Reduction by High-Temperature Gas-Source Method. Mater. Sci. Semicond. Process..

[12] Chen Q.-S., Zhang H., Ma R.-H., Prasad V., Balkas C.M., Yushin N.K. (2001). Modeling of Transport Processes and Kinetics of Silicon Carbide Bulk Growth. J. Cryst. Growth.

[13] Calabretta C., Scuderi V., Bongiorno C., Cannizzaro A., Anzalone R., Calcagno L., Mauceri M., Crippa D., Boninelli S., La Via F. (2022). Impact of Nitrogen on the Selective Closure of Stacking Faults in 3C-SiC. Cryst. Growth Des..

[14] Liu H., Li K., Zhang X., Liu B., Qi L., Yin X. (2023). Controllable Growth of High-Density Tapered N-Doped SiC Nanowires Arrays. Ceram. Int..

[15] Tang Z., Gu L., Ma H., Dai K., Luo Q., Zhang N., Huang J., Fan J. (2023). Study on the Surface Structure of N-Doped 4H-SiC Homoepitaxial Layer Dependence on the Growth Temperature and C/Si Ratio Deposited by CVD. Crystals.

[16] Xuan L., Tong Z., Lu S., Wang A., Cui C., Huang Y., Deng T., Pi X., Yang D., Han X. (2025). Comparison of Resistivity Distribution in P-Type and n-Type SiC Single Crystals Grown by the PVT Method. Cryst. Eng. Comm..

[17] Inoue Y., Tochizaki W., Iwai T., Tanabe K., Ohtani N. (2024). Nitrogen Doping Concentration Dependence of Nitrogen Incorporation Kinetics during Physical Vapor Transport Growth of 4H–SiC Crystals. Mater. Sci. Semicond. Process..

[18] Liu X., Zhang J., Xu B., Lu Y., Zhang Y., Wang R., Yang D., Pi X. (2022). Deformation of 4H-SiC: The Role of Dopants. Appl. Phys. Lett..

[19] Fischer B., Nuys M., Haas S., Thimm O., Schöpe G., Foucart P., Besmehn A., Rau U. (2025). Synthesis and Characterization of Nitrogen-Doped Crystallized SiC Films from Liquid Precursors. ACS Appl. Electron. Mater..

[20] Carter S.G., Tathfif I., Burgess C., VanMil B., Debata S., Dev P. (2025). Influence of Nitrogen Doping and Annealing on the Silicon Vacancy in 4 H-SiC. Phys. Rev. B.

[21] Wu K., Mei Q., Liu H., Zhou S., Gao B., Li C., Liu S., Wan L. (2023). Vapor Deposition Growth of SiC Crystal on 4H-SiC Substrate by Molecular Dynamics Simulation. Crystals.

[22] Ha M.-T., Jeong S.-M. (2022). A Review of the Simulation Studies on the Bulk Growth of Silicon Carbide Single Crystals. J. Korean Ceram. Soc..

[23] Tang Z., Zhao S., Li J., Zuo Y., Tian J., Tang H., Fan J., Zhang G. (2024). Optimizing the Chemical Vapor Deposition Process of 4H–SiC Epitaxial Layer Growth with Machine-Learning-Assisted Multiphysics Simulations. Case Stud. Therm. Eng..

[24] Gallou Y., Hassant C., Potier A., Chaussende D. (2025). Thermal Modelling of a Hot-Wall CVD Reactor: Pressure and Composition of the Gas Phase in the Insulation Felt Matters. Vacuum.

[25] Haoxiang W., Renke K., Zhigang D., Shang G.A.O. (2025). Ultraprecision Machining for Single-Crystal Silicon Carbide Wafers: State-of-the-Art and Prospectives. J. Adv. Manuf. Sci. Technol..

[26] Chen Y., Wu H., Zhang H., Gao A., Gong L., Zeng S., Li S., Liu M., Li Q. (2025). Preparation of Carbon/Silicon Carbide Composites Based on Selective Laser Sintering/Reaction Melt Infiltration and Its Theoretical Modeling Calculations. J. Mater. Res. Technol..

[27] Gong X., Li P., Xie T., Hu F., Ba S., Wang L., Zhu W. (2024). High Uniform N-Type Doping of 4H-SiC Homoepitaxy Based on a Horizontal Hot-Wall Reactor. J. Cryst. Growth.

[28] Singh A., Mahamiya V., Shukla A. (2023). Defect-Driven Tunable Electronic and Optical Properties of Two-Dimensional Silicon Carbide. Phys. Rev. B.

[29] Kaloyeros A.E., Arkles B. (2023). Silicon Carbide Thin Film Technologies: Recent Advances in Processing, Properties, and Applications-Part I Thermal and Plasma CVD. ECS J. Solid State Sci. Technol..

